# Practice-based analysis of direct posterior dental restorations performed in a public health service: Retrospective long-term survival in Brazil

**DOI:** 10.1371/journal.pone.0243288

**Published:** 2020-12-22

**Authors:** Renata Afonso da Silva Pereira, Gisele Rodrigues da Silva, Luciana Mendes Barcelos, Karoline Guará Brusaca Almeida Cavalcanti, Álex Moreira Herval, Thiago Machado Ardenghi, Carlos José Soares

**Affiliations:** 1 Department of Operative Dentistry and Dental Materials, School of Dentistry, Federal University of Uberlândia, Uberlândia, Minas Gerais, Brazil; 2 Department of Postgraduate Program in Dentistry, CEUMA University, São Luis, Maranhão, Brazil; 3 Department of Social and Preventive Dentistry, School of Dentistry, Federal University of Uberlândia, Uberlândia, Minas Gerais, Brazil; 4 Department of Stomatology, Federal University of Santa Maria, Santa Maria, Rio Grande do Sul, Brazil; Danube Private University, AUSTRIA

## Abstract

The aim of this retrospective study was to evaluate the survival and associated factors for the longevity of direct posterior restorations and to verify whether the geographic location of public health units could influence the long-term survival of such restorations. Data were extracted from electronic patient files of the Brazilian public oral health services. The sample comprised 2,405 class I and II restorations performed 4 to 24 years ago (mean, 8.9 years) in 351 patients (6.8 teeth/patient) across 12 public health units located in different city regions (42 professionals—55 restorations). The restoration was considered successful if it had not been repaired or replaced at the time of evaluation; failure was defined as replacement of the restoration, the need for endodontic treatment, tooth/restoration fracture or tooth extraction. Data were analyzed using the Kaplan-Meier test for restoration survival and Cox regression to evaluate the factors associated with failure. The majority of the restorations involved the use of amalgam (85%), involved a single face (70%), and were without pulp/dentin capping (85%). The overall survival rate was 95%, and the mean observation time was 8.9 years. The restoration survival was 79% (95% CI: 60.6–89.5) over 24 years, and the mean survival time was 22.2 years (95% CI: 21.9–22.6 years). The annual failure rate up to 24 years was 0.9%. After the adjustment, only the number of restored faces and the geographic location where the restoration was performed remained associated with failure of the restoration. The direct posterior restorations performed at the evaluated public health service units presented high survival rates. The restorations of people with lower access to POHS had lower survival rates. Class I restorations presented higher survival rates than class II restorations with two or more faces, regardless of the restorative material used.

## Introduction

Direct restoration is the prevalent dental health service performed in both private and public clinics in the majority of developed and developing countries [1]. These treatments represent important financial issues for patients and health care systems, especially if they fail to require the replacement [2]. Despite the decrease in caries prevalence in many countries, there is still a high need for posterior restorative treatment [3].

In general, for decades, amalgam has been the first-choice material for posterior tooth restoration [4]. The Minamata Convention encouraged alternatives to amalgam restorations, stimulating the use of resin composite [5, 6] and highly viscous, glass-ionomer cement/resin-coated restorations [7, 8]. However, clinicians often prefer to use amalgam restoration in a public service unit due to the conditions under which the restorations are performed and because amalgam is thought to have better longevity than resin composite [6]. Resin composite materials have been gaining popularity due to their aesthetics and adhesive properties and are overtaking amalgam [9]. Significant improvements have been made in the physical properties of resin composites, and they are currently the choice of dentists for restorative material for different clinical applications [10]. Additionally, there is low-quality evidence to suggest that resin composites lead to higher failure rates and a higher risk of secondary caries than amalgam restorations [11, 12].

The option to select resin composite or amalgam in several countries has been determined by covering the treatment under health insurance or public service rules [3]. The Unified Health System (*Sistema Único de Saúde*—SUS) is one of the largest free-of-charge methods to access health actions and services in the world [13]. This public health system benefits approximately 75% of Brazilian people with health care, including procedures from simple to highly complex [13, 14]. The implementation of public oral health services (POHS), a strategy called "Smiling Brazil", has been an important improvement for the SUS [13].

The decision to choose amalgam or resin composite as part of POHS is not determined by cost or previous determination of SUS benefits. Currently, large databases of insurance companies and public health service units facilitate retrospective analyses of outcome data that can represent clinical reality [1], serving as an evaluation instrument to investigate and improve the establishment of services for the population. The performance of practice-based research is highly recommended and can present a great contribution to decision-making for the restorative material selection [15]. Providing accurate information on restoration survival is relevant for improving the quality of public oral health assistance [1, 3]. The failure of posterior restoration is not only related to material properties but also dependent on the patient’s habits and the operator’s performance [3].

The correlations among the profiles of the clinicians, the education level of the patients and their access to oral health services and the material selected to restore posterior teeth in a public service unit may affect the clinical performance [15]. Therefore, the aim of this document-based retrospective study was to evaluate the longevity of posterior restorations according to the type of tooth, size of the restoration, restorative material used, and characteristics of the dentists who performed the procedure in Brazilian public health service units. The null hypothesis of the study was that the geographic location of the POHS units and the education level of the patients would not influence the longevity of the long-term survival of these restorations.

## Materials and methods

### Study design

This retrospective, practice-based study was performed at the POHS units of the city of Uberlândia, Minas Gerais (MG), Brazil, to evaluate the longevity of direct posterior restorations. Data were extracted from medical records of the POHS of the SUS. All restorations were performed by the primary health care clinicians. The medical records were reviewed during the period from August to December 2019, and restorations performed between January 1986 and January 2015 were evaluated.

#### Inclusion criteria

Patients visited the oral health service department of the primary health care unit of Uberlândia, MG, Brazil. Patients visited one of 12 public primary health care units distributed in 5 regions of the urban area (center, north, south, east and west); these units employed 42 professionals who performed at least 10 restorations each and had systematic patient dental/medical files. Patients who received at least one posterior direct restoration made with amalgam or resin composite in a permanent vital tooth and who had returned annually/biannually for examinations for a minimum of 5 years were included in the study.

#### Exclusion criteria

The exclusion criteria were as follows: patients who received restorations performed with compomers, zinc phosphate cement, or zinc eugenol cement; patients who received restorations performed in deciduous teeth or endodontic treated teeth; and patients who did not return annually for clinical evaluations for at least 5 years.

### Ethical considerations

Our data are based on patient documents from the database of the POHS of Uberlândia city. The original recordings were made at each appointment. Before providing the data, the patient identifiers were removed and replaced by consecutive numbers to ensure confidentiality. The ethical committee of the Federal University of Uberlândia approved the study protocol (CAAE: 57908016.8.0000.5152) on July 26, 2018. The Department of Social Services and Healthcare of the city of Uberlandia approved the study.

### Restorations evaluated

A total of 2,405 class I and II restorations made in vital teeth 5 to 29 years ago (mean, 8.9 years) in 351 patients (6.8 teeth/patient) comprised our final sample. A total of 836 medical records were evaluated; 485 records were excluded using the listed criteria, and the medical records were defined using a systematic sample.

Two experienced clinicians were previously trained and calibrated regarding the methods and assessment of the oral health records, which involved all of the following restoration aspects: gender of patients (male or female); material used for each restoration (amalgam or resin composite); date of placement; date of last intervention; date of last checkup; tooth type (premolar or molar); tooth arc (mandibular or maxilla); the number of restoration faces (1 or 2 and more); use of pulp/dentin capping (yes or no); public health unit location in the city region (center, east, north, west, south); gender of the professional who performed the restoration (male or female); professional with specialization (yes or no); graduation time (less than 20 or more than 20 years); and condition of the restoration (failure or success).

Restorations of the posterior teeth were considered clinically functional and aesthetically successful if they were not repaired or replaced within the minimal survival period of 4 years. The following outcomes were considered as clinical failure: restoration failure with the teeth submitted to indirect rehabilitative treatment, repair or replacement of the restoration, endodontic treatment, fractures of the teeth and restorations leading to dental extraction [16].

### Statistical analysis

The extracted data were compiled into statistical software package STATA 14.0 (Stata 14.0 for Windows; Stata Corporation, College Station, TX, USA) to be analyzed. The data analysis included descriptive statistics of the main features of patients and restored teeth as well as the ratings of restoration outcomes. The annual failure rate (AFR) of the restorations was calculated according to the following formula: (1− y)^z^ = (1− x). In this formula, “y” expresses the mean AFR, and “x” represents the total failure rate at “z” years. The longevity of the restorations was evaluated through the survival analysis using the Kaplan-Meier method. Differences in survival rates according to the studied variables were evaluated through the multivariate Cox regression analysis with shared frailty (restorations clustered in patients). Variables presenting *P* values < 0.20 in the unadjusted analysis were entered into the multivariate model. For all hypothesis tests, the statistical significance was set at α = 0.05 after the adjustments.

## Results

Patient gender, the identified characteristics of restored teeth, restorative material, the use of pulp/dentin capping, professionals’ profile, and location where the restorations were performed are shown in Table 1.

**Table 1:** Status of restorations according to clinical and sociodemographic characteristics (351 patients; n = 2,405 restorations), Uberlândia, MG, Brazil.

Variable		Success		Failure	
		n	%	n	%
Patient gender	Female	1,552	94.8	86	5.2
	Male	731	95.3	36	4.7
Tooth type	Molar	1,437	94.5	83	5.5
	Premolar	846	95.6	39	4.4
Tooth arch	Maxilla	1,146	95.5	54	4.5
	Mandibula	1,137	94.4	68	5.6
Restorative material	Amalgam	1,941	95.0	102	5.0
	Resin composite	342	94.5	20	5.5
Number of restoration faces	1 face	1,586	95.4	77	4.6
	2 or more faces	697	93.9	45	6.1
Use of pulp/dentin capping	Yes	1,944	95.2	98	4.8
	No	339	93.4	24	6.6
City region of public health unit	Center	196	98.0	4	2.0
	East	272	97.8	6	2.2
	North	337	89.6	39	10.4
	West	1,013	93.5	70	6.5
	South	465	99.4	3	0.6
Professionals with specialization	Yes	1,629	95.3	81	4.7
	No	654	94.1	41	6.0
Graduation time	≤ 20 years	665	99.1	6	0.9
	> 20 years	1,618	93.3	116	6.7

N: number of occurrences; %: percentage.

The patients who visited the 12 public health units were predominantly women, without systemic alterations. The restorations were preferentially made of amalgam, involving a single face; the majority were without pulp/dentin capping. The vast majority of clinicians were women, with specializations and approximately 20 years of training. Restorations performed in a unit of the POHS in the north and west regions had higher percentages of failure than those performed in the south, east and center regions. Compared to the other regions, the north and west regions also had a lower coverage of dental care.

**Fig 1:**
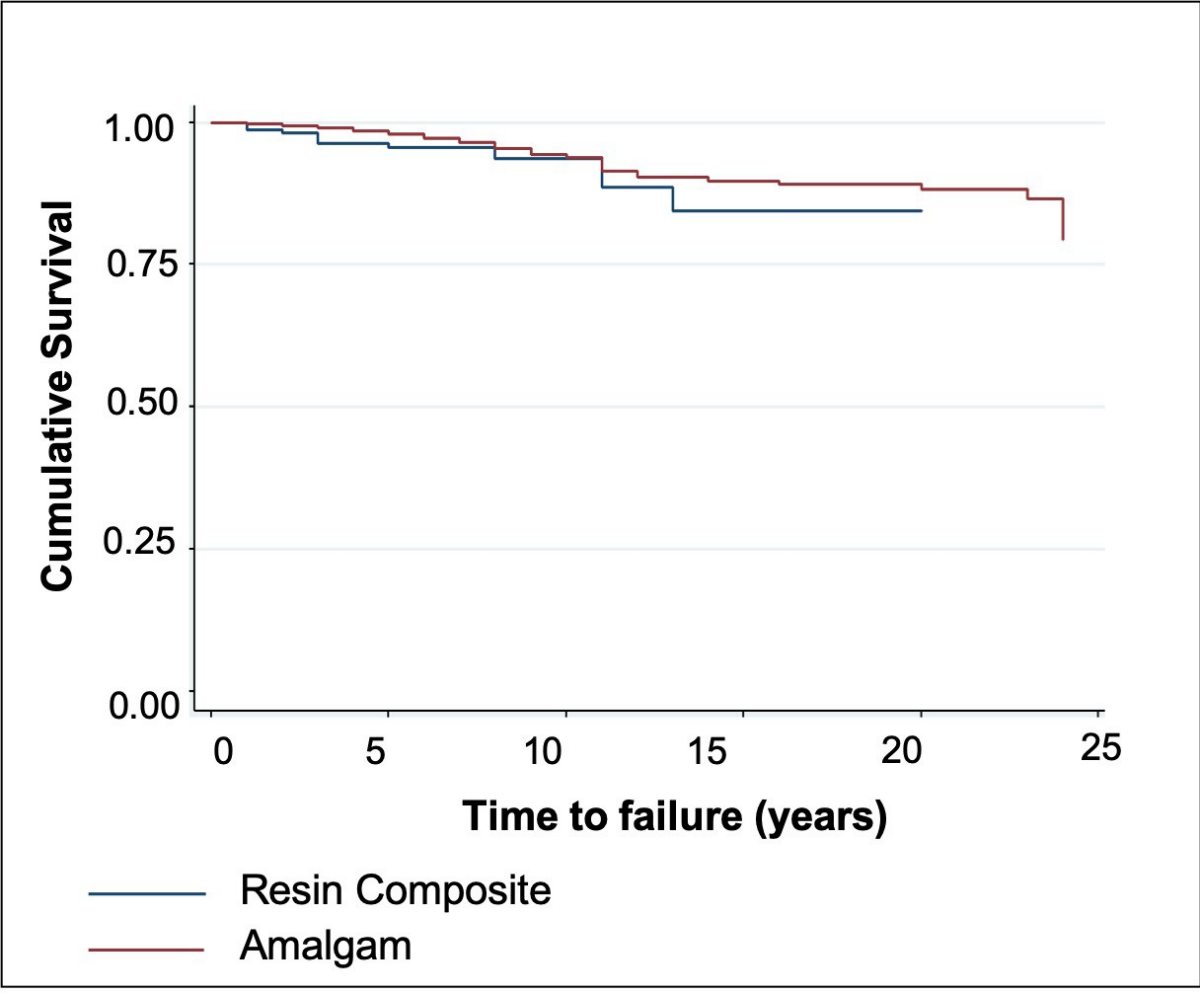
Kaplan-Meier plots comparing amalgam and resin composite subgroups. The *P* values indicate no significant differences between subgroups. Survival times were censored if the event (i.e., failure of the restoration) did not occur during the follow-up period (blue line for amalgam restorations; red line for resin composite restorations).

The cumulative restoration survival estimates are shown in Fig 1. The total number of failures was 122 (5.1%), and the mean observation time was 8.9 years. The survival of the restoration reached 79% (95% CI: 60.6–89.5) up to 24 years, and the mean survival time was 22.2 years (95% CI: 21.9–22.6 years). The annual failure rate up to 24 years was 0.9%. No significant difference in survival was found between amalgam or resin composite (Fig 1).

Unadjusted and adjusted hazard ratios (HR) for the restorations according to the clinical and sociodemographic characteristics are shown in Table 2.

**Table 2:** Unadjusted and adjusted hazard ratios (HR) for independent variables and failure of the restorations (351 patients; n = 2,405 restorations). Cox regression analysis with shared frailty, Uberlândia, MG, Brazil.

Variable		HR_unadjusted_ (95% CI)	*P*-value	HR_adjusted_ (95% CI)*	*P*-value
Patient gender	Male	1			
	Female	1.11 (0.60–2.11)	0.75		
Tooth type	Molar	1			
	Premolar	0.81 (0.54–1.21)	0.29		
Tooth arch	Mandibula	1		1	
	Maxilla	0.76 (0.52–1.10)	0.15	0.71 (0.49–1.04)	0.08
Restorative material	Resin composite	1		1	
	Amalgam	0.61 (0.35–1.07)	0.08	0.57 (0.32–1.01)	0.053
Number of restoration faces	1 face	1		1	
	2 or more faces	1.52 (1.00–2.33)	0.05	1.61 (1.04–2.49)	0.03
Use of pulp/dentin capping	No	1			
	Yes	0.99 (0.56–1.76)	0.99		
City region of public health unit	North	1		1	
	South	0.04 (0.00–0.16)	0.00	0.07 (0.01–0.37)	0.00
	West	0.24 (0.08–0.75)	0.02	0.71 (0.08–6.36)	0.76
	East	0.28 (0.15–0.52)	0.00	0.28 (0.15–0.53)	0.00
	Center	0.14 (0.04–0.52)	0.00	0.17 (0.04–0.67)	0.01
Professionals with specialization	No	1			
	Yes	0.88 (0.49–1.57)	0.66		
Graduation time	≤ 19 years	1		1	
	> 19 years	4.12 (1.55–10.89)	0.00	3.16 (0.41–24.36)	0.270

*Variables were included in the adjusted model only if they had a *P*-value < 0.20 in the unadjusted analysis.

No significant difference in the survival rates of the restorations was found between genders. Specialists had failure rates similar to those of general clinicians. The use of a pulp/dentin capping material had no influence on the survival rate of posterior restorations. Restorations with more than one face were more likely to result in failure than single face restorations (HR: 1.52)

**Fig 2:**
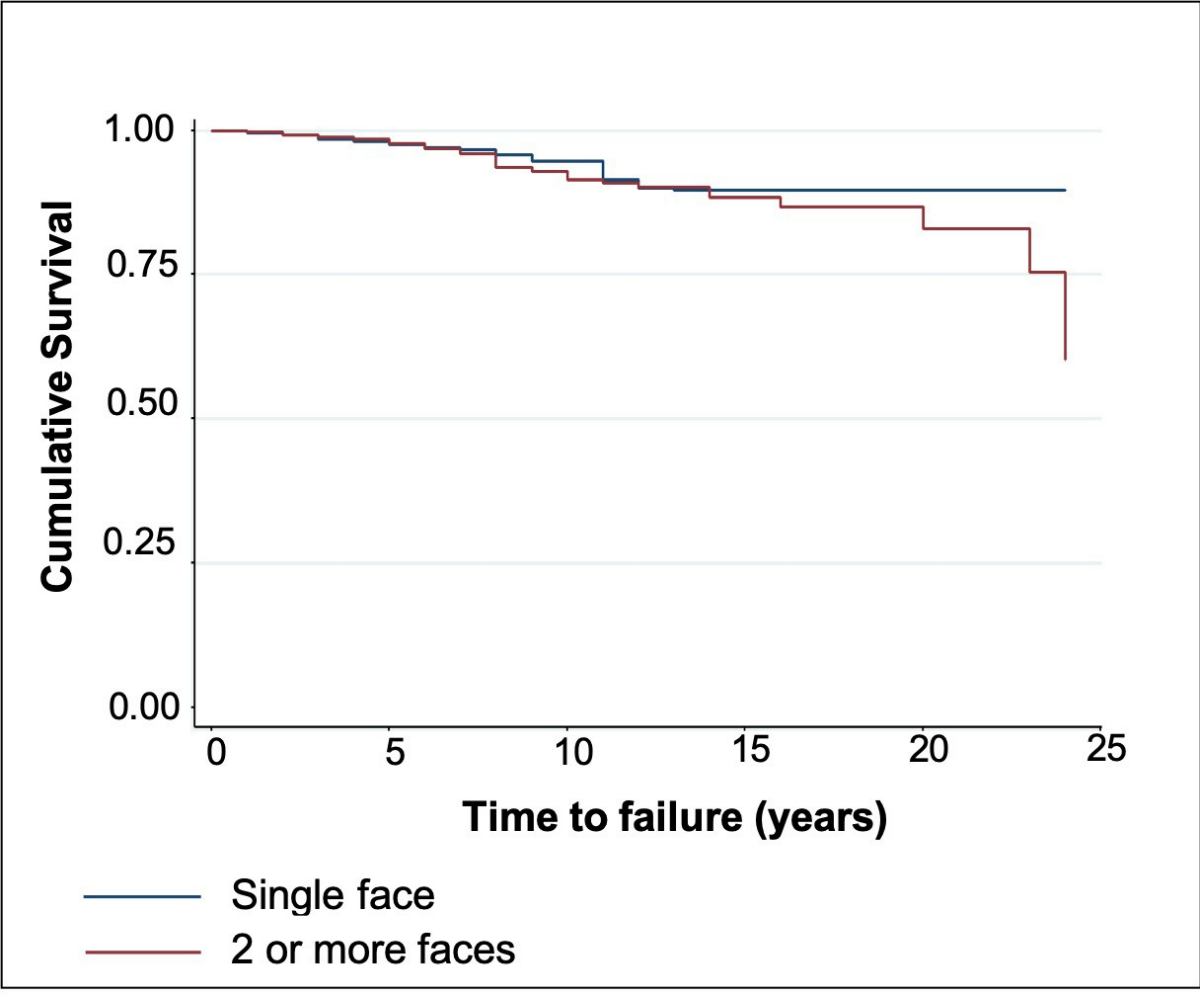
Kaplan-Meier plots comparing single and multiple surfaces. Survival times were censored if the event (i.e., failure of the restoration) did not occur during the follow-up period (blue line for single face restorations; red line for multiple face restorations).

**Fig 3:**
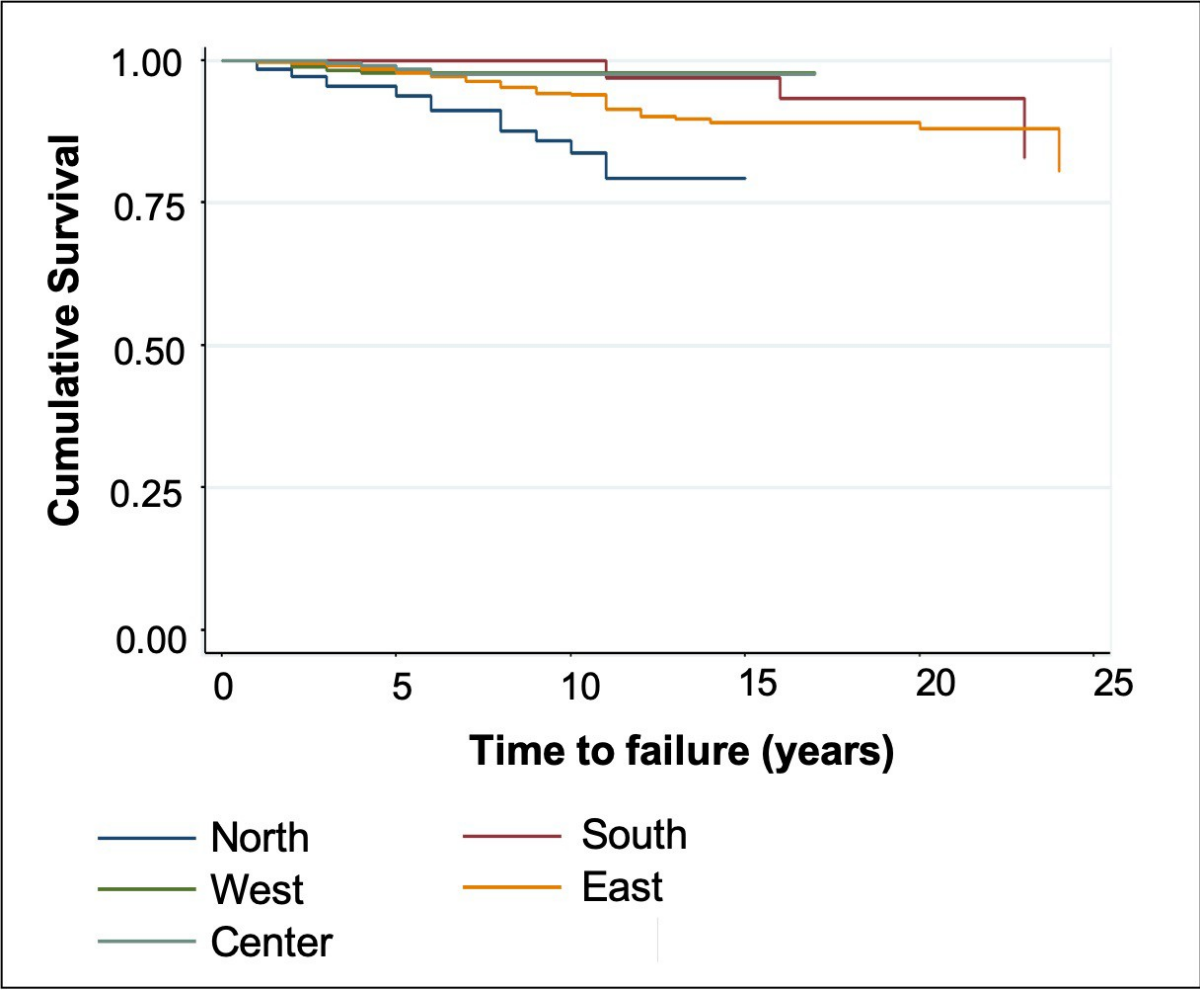
Kaplan-Meier plots comparing the geographic location where the restorations were performed. Survival times were censored if the event (i.e., failure of the restoration) did not occur during the follow-up period.

Restorations performed by professionals who graduated within the past 19 years were less likely to result in failure than those performed by professionals who graduated more than 19 years ago (HR: 4.12). A significant difference was observed across geographic locations where the restorations were performed, with the north and west regions having the highest failure rate (*P* < 0.001). After adjustment, only the number of restored faces and the geographic location where the restoration was performed remained associated with failure of the restoration. Compared with single face restorations, those with more than two faces presented a higher HR. Notwithstanding, restorations performed in the east, south and center regions were less likely to result in failure than those performed in the north and west regions. Figs 2 and 3 demonstrate the cumulative survival charts according to the number of faces and geographic location, respectively.

The failed restorations were frequently replaced using the same material used during the first intervention. The indication of pulp/dentin capping increased by approximately 6% for amalgam and 25% for resin composite restorations. The cavity, expressed by the number of faces, was extended by 18% for amalgam and 30% for resin composite, which is shown in Table 3.

**Table 3:** Characteristics of the failed restorations regarding restorative material, number of restorative surfaces before and after reintervention, use of pulp/dentin capping before and after reintervention, material used for reintervention, and decision-making for reintervention.

Variable		Failures			
		Amalgam		Resin Composite	
		N.	%	N.	%
		102	5.0	20	5.5
Decision-making for reintervention	Repair	23	22	4	20
	Replacement	79	78	16	80
Material used for reintervention	Maintained	78	76	12	60
	Changed	24	24	8	40
Use of pulp/dentin capping	Yes	23	22	1	5
	No	79	78	19	95
Use of pulp/dentin capping after reintervention	Yes	29	28	6	30
	No	73	72	14	70
Number of restoration faces	1 face	59	58	18	90
	2 or more faces	43	42	2	10
Number of faces after reintervention	Maintained	84	82	14	70
	Extended	18	18	6	30

N: number of occurrences; %: percentage.

The characteristics of the professionals who worked at the 5 public health units involved in this study are shown in Table 4. The public health unit in the west region had a higher number of professionals and restorations performed. However, the mean numbers of restorations performed by professionals in different public health units were very similar.

**Table 4:** Characteristics of professionals and restorations performed per public health unit.

City region of public health unit	N	%	Number of professionals	Mean (SD) age of professionals	Number of restorations/professional
Center	200	8.3	4	24 (5.5)	50
East	278	11.6	5	20 (4.7)	56
North	376	15.6	7	31 (6.8)	60
West	1.083	45.0	18	28 (6.5)	60
South	468	19.5	9	22 (4.5)	59

## Discussion

The null hypothesis of this retrospective, practice-based study was rejected, as the geographic location of the POHS unit influenced the long-term survival of both amalgam and resin composite direct posterior restorations. Personal (gender) and restorative variables (tooth type, tooth arc, restorative material, and use of pulp/dentin capping) were also associated with the failure of direct posterior restorations. However, after adjustment, only the number of restored faces was associated with failure of the restoration. Compared with single face restorations, those with more than two faces presented a higher failure rate.

The geographic locations of the POHS units with the highest frequencies of restoration failure were also the regions with the lowest population coverages of the POHS, with limited access of the population to oral health care. Adequate treatment coverage is an important task to prevent secondary caries, which is the most common reason for restoration failure [17]. A study performed in the United States showed that children covered by public dental services were more likely to have preventive dental care than children linked to private dental services, even when the statistical model was adjusted for family income [18]. Oral health has been considered a part of integral health, and Brazilian policies stipulate the inclusion of dentists in primary health care, as the largest part of the population covered by primary care remains without the coverage of oral health professionals [19]. This reality has been aggravated by the Brazilian economic crisis and by austerity measures, which are reducing POHS coverage in primary health care [20]. The low coverage of the POHS highlights the inequity of access to health services. Some determinants of these inequalities can be identified and include geographic and social inequalities in the supply of health care services, individual lifestyle factors, social and community networks, and socioeconomic, cultural and environmental conditions [19]. Furthermore, the use of dental services is influenced by the availability of those services, including the geographic distribution of dentists as well as the resources of the health service that fit the needs of the community [21]. Unfortunately, disadvantaged populations are also higher in the north and west regions, which may explain the lower survival rate of the posterior restorations. This information is important for public health policy and can contribute to governing strategies to prioritize the investment in populations with an inequity of access to POHS.

The longevity of direct posterior resin composite restorations is well established for permanent teeth [22]. There are aspects that can significantly influence the survival rate of resin composite restorations, such as the extension of decay leading to the size of the cavity and occlusal problems [23, 24]. Restoration replacement is one of the most common dental procedures in public and private dental offices, representing a high financial cost for individuals and for the public health system [5, 6].

The ‘National Program for Improving Access to and Quality of Primary Care’ in Brazil evaluated the southeast region (5,027 dental teams) between 2013 and 2014 and showed that 98.4% of the dental teams performed resin composite restorations and 93.5% performed amalgam posterior restorations [25, 26]. In the past 25 years, there has been a steady growth in the use of resin composite materials for the restoration of posterior teeth. The new generation of professors who are joining the teaching process with more experience in performing posterior resin composite restorations may also have contributed to the changes in resin composites used for restorations in posterior teeth, especially because of the preference for aesthetic materials [27]. Certainly, it is sensible to assume that the change assimilated within the university will be further reflected in the choices the young graduates will make [7]. Professionals who graduated recently preferred resin composite for posterior restorations in the majority of cases, in contrast to more experienced university members, who preferred amalgam [7]. The prevalence of amalgam restoration performed in the evaluated public service units can be related to the graduation time of the clinicians who work in this heath system. The job stability in the evaluated public services reflected the continued activity at the same location and made possible the correlation of the restoration performance with the clinicians’ profiles. The teaching process received by most clinicians was, only or prevalently, performing amalgam restorations. Additionally, structural, sociocultural, and familial factors can impact the ability of the population selected in this study to utilize oral care services. The education level of patients and parents can impact autonomy in treatment options [28]. The combination of these factors with the paradigm that amalgam performed better than resin composite, which was the response of all of the oldest clinicians, may contribute to the material choice during posterior resin composite restoration. The difference in the amalgam and resin composite restoration ratios observed in this study can have an impact on the real effect conditional parameters evaluated, which can be considered a limitation of this study.

The indication of amalgam has been questionable, even in a public health setting; however, the findings of this study suggest that amalgam restorations that are performed properly should not be removed only to be replaced by resin composite. It is imperative that practitioners be made aware of the global changes in the guidelines pertaining to the handling and disposal of amalgam, to include dental amalgam safety protocols, and the benefits of mercury-free practices should be made a part of the existing academic curriculum [29]. It is not recommended to replace dental amalgam fillings as long as they are in good condition. Drilling out amalgam will induce the removal of healthy tooth structure, and the process will expose the patient and the ambient air to considerably more mercury vapor than if the restoration was maintained.

Accordingly, in this study, the professionals’ ages and graduation times had significant effects on restoration survival. A possible cause is that younger dentists who recently graduated might still be following the teaching practices from dental school. However, the use of the most up-to-date techniques is usually by younger dentists, who may have been trained to adopt a more cautious, ‘wait and see’ approach [7]. An important aspect observed in this study was that the city region of the primary health care unit that had a lower percentage of restoration failures was concentrated in units in which professionals had the shortest training time. The main reason is most likely the increased awareness of the advantage of a minimally invasive approach in treating decays [8]. The high rate of class I cavities involved in this study should be considered since the higher caries risk in adults and adolescents is on proximal surfaces. This aspect can be related to the equivocated diagnosis of occlusal caries [30]. Occlusal discoloration as a diagnostic criterion could lead to a false-positive caries diagnosis and, consequently, the premature indication of restorative procedures, assuming that placing a restoration “fixes” dental caries, which is not true [31]. Another aspect that can reflect the high rate of class I cavities is due to the absence of radiography equipment in these public health units. The principal means employed to diagnose occlusal caries are visual and tactile; for proximal caries, it is bitewing radiography [32, 33]. Consequently, without the equipment for diagnosis, fewer interventions are performed.

The present study showed no statistical influence of the position of the tooth in the arch. Similar results were observed in previous studies that showed similar performance for the restorations performed in molar and premolar teeth [17, 34]. Other studies have reported that restorations in the mandibular arch perform less favorably than those in the maxillary arch [15]. The number of faces of the restorations was associated with failure. Several studies have demonstrated that multiple surface restorations perform worse than single surface restorations [15, 16, 35]. In this study, after the adjustment, the number of restored faces was associated with restoration failure. Compared with single face restorations, those with more than two faces presented a higher HR. Two- and three-surface resin composite restorations are considered the cavities with the highest risk of developing shrinkage stress [35], and two- and three-surface amalgam restorations can be more susceptible to fracture caused by weakened tooth structure and the absence of the adhesive integration [2, 36]. Our results agree with another study that found no significant difference regarding the restorative material in relation to cavity size [37].

The annual failure rate percentage over 24 years was 0.9%, with similar performances for amalgam and resin composite. The 24-year survival of the restorations reached 79%. The performance of the restorations carried out in the public health service units evaluated in this study was similar to that in other studies [38–40]. A systematic review found a rate of 1.55% of failures per year for posterior resin composite restorations [38]. This result corresponded to a four-year survival rate of approximately 94%, which shows that survival rates in different risk groups were inferior but were still over 90% at 4 years [39]. Another prospective trial from the Danish public health service that included 4355 restorations with an observation period of more than 8 years showed a favorable 84% eight-year survival rate [42].

Many patient and dentist-related factors influence the survival of dental restorations [41]. A prospective study of a Swedish public health service that included 63 molars revealed a survival rate of 72% at 5 years, which was inferior to other general practice data [42]. An important aspect observed in the current practice-based study was the decision-making for intervention options defined by professionals; when either type of restoration failed, most were replaced instead of repaired. Most general dental practitioners choose replacement as opposed to systematic restoration monitoring or repair [43, 44].

This study presented some limitations. Large population studies have typically relied on claims databases or publicly available national databases. However, these databases often lack point-of-care data collected at actual clinic visits. Regarding the inclusion of one city to represent the Brazilian public health system, Uberlândia is one of the municipalities with the best average indicators regarding per capita income, formalization of the labor market, educational indicators and vulnerability in Brazil [45]. However, the great sociocultural and educational variability observed in different locations of the municipality make this selected city an adequate scenario to represent the entire country. It was impossible to individually record the information of each restoration regarding the size of the cavity and the adhesive systems and resin composite used, which could better define the quality of the restorative procedures. Regardless of these limitations, the posterior restorations performed in the POHS of Uberlândia showed high survival rates for both resin composite and amalgam, expressing the quality of service offered. The two restorative options are related to the most commonly used restorative protocols performed in this public health system. To properly support the phase-down of dental amalgam in the public health system, it is necessary to consider merging low-cost alternative protocols that are minimally invasive, such as highly viscous glass-ionomer cement/resin composite coating restorations [7, 8, 46], which might be a restorative option for patients with financial limitations. The findings of this study are important for public services, not only in Brazil or developing countries, for designing better strategies for improving oral health practices. This study can speculate that for improving the longevity of posterior restorations, better qualification of the professionals, including education at work programs, and increasing the POHS coverage mediated by the promotion of equity in the access to health services would be beneficial.

## Conclusions

In this study, it is possible to conclude that both amalgam and resin composite restorations in posterior teeth performed in public health service units presented similarly high survival rates. The longevity of the restorations was influenced by the geographical location of the clinics, the profile of the professionals regarding their graduation time, and the number of restored faces. The restorations of populations with lower access to POHS had lower survival rates, restorations performed by clinicians with more recent graduation times presented higher survival rates, and restorations with more than two faces presented a higher HR than single face restorations.

## Supporting information

S1 Data Database of all restorations, patients, professionals, and location where the restorations were performed. (XLS)

S2 Data Statistical analysis. (PDF)
