# Supplementary material for: Practice-based analysis of direct posterior dental restorations performed in a public health service: Retrospective long-term survival in Brazil
Source: PLoS One. 2020 Dec 22;15(12):e0243288. doi: 10.1371/journal.pone.0243288 (PMC7755217; doi:10.1371/journal.pone.0243288)
Supplement: S2 Data — (PDF) [file pone.0243288.s002.pdf]

```
-------------------------------------------------------------------------------------
--------------
      name:  <unnamed>
       log:  C:\Users\thiar\Documents\lenovo\Backup
05-02-2020\_disco_D\Arquivos_User\Documents\e
> statisticas para outros\carlos soares\carlos 06-08 do file.log
  log type:  text
 opened on:   6 Aug 2020, 16:46:56

. do "C:\Users\thiar\Documents\lenovo\Backup
05-02-2020\_disco_D\Arquivos_User\Documents\estatist
> icas para outros\carlos soares\carlos 6-08-20.do"

. ** descriptives- table 1***
. tab feminino Condicao_Restauracao, row

+----------------+
| Key            |
|----------------|
|   frequency    |
| row percentage |
+----------------+

           | Condicao_Restauracao
  feminino |         0          1 |     Total
-----------+----------------------+----------
      male |       731         36 |       767 
           |     95.31       4.69 |    100.00 
-----------+----------------------+----------
    female |     1,552         86 |     1,638 
           |     94.75       5.25 |    100.00 
-----------+----------------------+----------
     Total |     2,283        122 |     2,405 
           |     94.93       5.07 |    100.00 


. tab premolar Condicao_Restauracao, row

+----------------+
| Key            |
|----------------|
|   frequency    |
| row percentage |
+----------------+

           | Condicao_Restauracao
  Premolar |         0          1 |     Total
-----------+----------------------+----------
         0 |     1,437         83 |     1,520 
           |     94.54       5.46 |    100.00 
```

```
-----------+----------------------+----------
         1 |       846         39 |       885 
           |     95.59       4.41 |    100.00 
-----------+----------------------+----------
     Total |     2,283        122 |     2,405 
           |     94.93       5.07 |    100.00 


. tab arcosuperior Condicao_Restauracao, row

+----------------+
| Key            |
|----------------|
|   frequency    |
| row percentage |
+----------------+

      arco | Condicao_Restauracao
  superior |         0          1 |     Total
-----------+----------------------+----------
         0 |     1,137         68 |     1,205 
           |     94.36       5.64 |    100.00 
-----------+----------------------+----------
         1 |     1,146         54 |     1,200 
           |     95.50       4.50 |    100.00 
-----------+----------------------+----------
     Total |     2,283        122 |     2,405 
           |     94.93       5.07 |    100.00 


. tab Amalgama Condicao_Restauracao, row

+----------------+
| Key            |
|----------------|
|   frequency    |
| row percentage |
+----------------+

           | Condicao_Restauracao
  Amalgama |         0          1 |     Total
-----------+----------------------+----------
         0 |       342         20 |       362 
           |     94.48       5.52 |    100.00 
-----------+----------------------+----------
         1 |     1,941        102 |     2,043 
           |     95.01       4.99 |    100.00 
-----------+----------------------+----------
     Total |     2,283        122 |     2,405 
           |     94.93       5.07 |    100.00 
```

```
. tab N_de_faces Condicao_Restauracao, row

+----------------+
| Key            |
|----------------|
|   frequency    |
| row percentage |
+----------------+

           | Condicao_Restauracao
N_de_faces |         0          1 |     Total
-----------+----------------------+----------
         0 |     1,586         77 |     1,663 
           |     95.37       4.63 |    100.00 
-----------+----------------------+----------
         1 |       697         45 |       742 
           |     93.94       6.06 |    100.00 
-----------+----------------------+----------
     Total |     2,283        122 |     2,405 
           |     94.93       5.07 |    100.00 


. tab RealizaçãodeForramento Condicao_Restauracao, row

+----------------+
| Key            |
|----------------|
|   frequency    |
| row percentage |
+----------------+

         1 |
  Realizaç |
    ão de  | Condicao_Restauracao
Forramento |         0          1 |     Total
-----------+----------------------+----------
         0 |       339         24 |       363 
           |     93.39       6.61 |    100.00 
-----------+----------------------+----------
         1 |     1,944         98 |     2,042 
           |     95.20       4.80 |    100.00 
-----------+----------------------+----------
     Total |     2,283        122 |     2,405 
           |     94.93       5.07 |    100.00 


. tab Setor_da_Cidade0 Condicao_Restauracao, row
```

```
+-----------------+
| Key             |
|-----------------|
|   frequency     |
| row percentage  |
+-----------------+

Setor_da_C | Condicao_Restauracao
    idade0 |         0          1 |     Total
-----------+----------------------+----------
     Norte |       337         39 |       376 
           |     89.63      10.37 |    100.00 
-----------+----------------------+----------
       sul |       465          3 |       468 
           |     99.36       0.64 |    100.00 
-----------+----------------------+----------
     leste |       272          6 |       278 
           |     97.84       2.16 |    100.00 
-----------+----------------------+----------
     oeste |     1,013         70 |     1,083 
           |     93.54       6.46 |    100.00 
-----------+----------------------+----------
    centro |       196          4 |       200 
           |     98.00       2.00 |    100.00 
-----------+----------------------+----------
     Total |     2,283        122 |     2,405 
           |     94.93       5.07 |    100.00 


. tab Espcialidade Condicao_Restauracao, row

+-----------------+
| Key             |
|-----------------|
|   frequency     |
| row percentage  |
+-----------------+

Espcialida | Condicao_Restauracao
        de |         0          1 |     Total
-----------+----------------------+----------
         0 |     1,629         81 |     1,710 
           |     95.26       4.74 |    100.00 
-----------+----------------------+----------
         1 |       654         41 |       695 
           |     94.10       5.90 |    100.00 
-----------+----------------------+----------
     Total |     2,283        122 |     2,405 
           |     94.93       5.07 |    100.00 
```

```
. tab temponovo Condicao_Restauracao, row

+----------------+
| Key            |
|----------------|
|   frequency    |
| row percentage |
+----------------+

   tempo de formado | Condicao_Restauracao
               <25% |         0          1 |     Total
--------------------+----------------------+----------
        até 19 anos |       665          6 |       671 
                    |     99.11       0.89 |    100.00 
--------------------+----------------------+----------
>19 anos de formado |     1,618        116 |     1,734 
                    |     93.31       6.69 |    100.00 
--------------------+----------------------+----------
              Total |     2,283        122 |     2,405 
                    |     94.93       5.07 |    100.00 


. ****cox regression with shared frailty- unadjusted***
. stcox feminino, shared (Numero_do_Paciente)

         failure _d:  Condicao_Restauracao
   analysis time _t:  Idade_da_restauracao

Fitting comparison Cox model:

Estimating frailty variance:

Iteration 0:   log profile likelihood = -826.44229
Iteration 1:   log profile likelihood = -826.19687
Iteration 2:   log profile likelihood = -826.19632
Iteration 3:   log profile likelihood = -826.19632

Fitting final Cox model:

Iteration 0:   log likelihood = -940.76142
Iteration 1:   log likelihood = -855.66978
Iteration 2:   log likelihood = -828.61691
Iteration 3:   log likelihood = -826.26161
Iteration 4:   log likelihood = -826.19678
Iteration 5:   log likelihood = -826.19632
Iteration 6:   log likelihood = -826.19632
Refining estimates:
Iteration 0:   log likelihood = -826.19632
```

```
Cox regression --
         Breslow method for ties                 Number of obs     =      2,405
         Gamma shared frailty                    Number of groups  =        348
Group variable: Numero_do_~e
                                                 Obs per group:
No. of subjects =        2,405                                 min =          1
No. of failures =          122                                 avg =  6.9109195
Time at risk    =        19386                                 max =         27

                                                 Wald chi2(1)      =       0.10
Log likelihood  =   -826.19632                   Prob > chi2       =     0.7496

------------------------------------------------------------------------------
          _t | Haz. Ratio   Std. Err.      z    P>|z|     [95% Conf. Interval]
-------------+----------------------------------------------------------------
    feminino |   1.107875   .3556207     0.32   0.750     .5905554    2.078361
-------------+----------------------------------------------------------------
       theta |   3.246451   .8064105
------------------------------------------------------------------------------
LR test of theta=0: chibar2(01) = 71.68               Prob >= chibar2 = 0.000

Note: Standard errors of hazard ratios are conditional on theta.

. stcox premolar, shared (Numero_do_Paciente)

         failure _d:  Condicao_Restauracao
   analysis time _t:  Idade_da_restauracao

Fitting comparison Cox model:

Estimating frailty variance:

Iteration 0:   log profile likelihood = -825.88287
Iteration 1:   log profile likelihood = -825.69953
Iteration 2:   log profile likelihood = -825.69939
Iteration 3:   log profile likelihood = -825.69939

Fitting final Cox model:

Iteration 0:   log likelihood = -939.75041
Iteration 1:   log likelihood = -854.49002
Iteration 2:   log likelihood = -828.03198
Iteration 3:   log likelihood = -825.76064
Iteration 4:   log likelihood = -825.69982
Iteration 5:   log likelihood = -825.69939
Iteration 6:   log likelihood = -825.69939
Refining estimates:
Iteration 0:   log likelihood = -825.69939

Cox regression --
```

```
         Breslow method for ties                Number of obs     =      2,405
         Gamma shared frailty                   Number of groups  =        348
Group variable: Numero_do_~e
                                                Obs per group:
No. of subjects =        2,405                                min =          1
No. of failures =          122                                avg =  6.9109195
Time at risk    =        19386                                max =         27

                                                Wald chi2(1)      =       1.08
Log likelihood  =   -825.69939                  Prob > chi2       =     0.2983

------------------------------------------------------------------------------
          _t | Haz. Ratio   Std. Err.      z    P>|z|     [95% Conf. Interval]
-------------+----------------------------------------------------------------
    premolar |   .8060494   .1670774    -1.04   0.298     .5369393    1.210036
-------------+----------------------------------------------------------------
       theta |   3.172394   .7957584
------------------------------------------------------------------------------
LR test of theta=0: chibar2(01) = 70.06             Prob >= chibar2 = 0.000

Note: Standard errors of hazard ratios are conditional on theta.

. stcox arcosuperior, shared (Numero_do_Paciente)

         failure _d:  Condicao_Restauracao
   analysis time _t:  Idade_da_restauracao

Fitting comparison Cox model:

Estimating frailty variance:

Iteration 0:   log profile likelihood = -825.44534
Iteration 1:   log profile likelihood = -825.18987
Iteration 2:   log profile likelihood = -825.18923
Iteration 3:   log profile likelihood = -825.18923

Fitting final Cox model:

Iteration 0:   log likelihood = -940.91041
Iteration 1:   log likelihood = -855.03981
Iteration 2:   log likelihood = -827.65698
Iteration 3:   log likelihood = -825.25547
Iteration 4:   log likelihood = -825.18971
Iteration 5:   log likelihood = -825.18923
Iteration 6:   log likelihood = -825.18923
Refining estimates:
Iteration 0:   log likelihood = -825.18923

Cox regression --
         Breslow method for ties                Number of obs     =      2,405
```

```
         Gamma shared frailty                   Number of groups  =        348
Group variable: Numero_do_~e
                                                Obs per group:
No. of subjects =        2,405                                 min =          1
No. of failures =          122                                 avg =  6.9109195
Time at risk    =        19386                                 max =         27

                                                Wald chi2(1)      =       2.10
Log likelihood  =   -825.18923                  Prob > chi2       =     0.1471

------------------------------------------------------------------------------
          _t | Haz. Ratio   Std. Err.      z    P>|z|     [95% Conf. Interval]
-------------+----------------------------------------------------------------
arcosuperior |   .7586327   .1445444    -1.45   0.147     .5222155    1.102081
-------------+----------------------------------------------------------------
       theta |   3.257461    .807853
------------------------------------------------------------------------------
LR test of theta=0: chibar2(01) = 72.09                Prob >= chibar2 = 0.000

Note: Standard errors of hazard ratios are conditional on theta.

. stcox Amalgama, shared (Numero_do_Paciente)

         failure _d:  Condicao_Restauracao
   analysis time _t:  Idade_da_restauracao

Fitting comparison Cox model:

Estimating frailty variance:

Iteration 0:   log profile likelihood =  -825.1005
Iteration 1:   log profile likelihood = -824.84512
Iteration 2:   log profile likelihood = -824.84447
Iteration 3:   log profile likelihood = -824.84447

Fitting final Cox model:

Iteration 0:   log likelihood = -940.92083
Iteration 1:   log likelihood = -854.66782
Iteration 2:   log likelihood = -827.26881
Iteration 3:   log likelihood =  -824.9097
Iteration 4:   log likelihood = -824.84493
Iteration 5:   log likelihood = -824.84447
Iteration 6:   log likelihood = -824.84447
Refining estimates:
Iteration 0:   log likelihood = -824.84447

Cox regression --
         Breslow method for ties                Number of obs     =      2,405
         Gamma shared frailty                   Number of groups  =        348
```

```
Group variable: Numero_do_~e
                                                Obs per group:
No. of subjects =        2,405                              min =          1
No. of failures =          122                              avg =  6.9109195
Time at risk    =        19386                              max =         27

                                                Wald chi2(1)      =       2.99
Log likelihood  =   -824.84447                  Prob > chi2       =     0.0838

------------------------------------------------------------------------------
          _t | Haz. Ratio   Std. Err.      z    P>|z|     [95% Conf. Interval]
-------------+----------------------------------------------------------------
    Amalgama |   .6132519   .1734398    -1.73   0.084     .3522916    1.067519
-------------+----------------------------------------------------------------
       theta |   3.258232   .8090491
------------------------------------------------------------------------------
LR test of theta=0: chibar2(01) = 71.27              Prob >= chibar2 = 0.000

Note: Standard errors of hazard ratios are conditional on theta.

. stcox N_de_faces, shared (Numero_do_Paciente)

         failure _d:  Condicao_Restauracao
   analysis time _t:  Idade_da_restauracao

Fitting comparison Cox model:

Estimating frailty variance:

Iteration 0:   log profile likelihood = -824.76944
Iteration 1:   log profile likelihood = -824.40894
Iteration 2:   log profile likelihood = -824.40708
Iteration 3:   log profile likelihood = -824.40708

Fitting final Cox model:

Iteration 0:   log likelihood =  -942.4014
Iteration 1:   log likelihood = -855.68071
Iteration 2:   log likelihood = -826.96877
Iteration 3:   log likelihood = -824.47702
Iteration 4:   log likelihood =  -824.4076
Iteration 5:   log likelihood = -824.40708
Iteration 6:   log likelihood = -824.40708
Refining estimates:
Iteration 0:   log likelihood = -824.40708

Cox regression --
         Breslow method for ties                Number of obs     =      2,405
         Gamma shared frailty                   Number of groups  =        348
Group variable: Numero_do_~e
```

```
                                                Obs per group:
No. of subjects =        2,405                               min =          1
No. of failures =          122                               avg =  6.9109195
Time at risk    =        19386                               max =         27

                                                Wald chi2(1)      =       3.80
Log likelihood  =   -824.40708                  Prob > chi2       =     0.0514

------------------------------------------------------------------------------
          _t | Haz. Ratio   Std. Err.      z    P>|z|     [95% Conf. Interval]
-------------+----------------------------------------------------------------
  N_de_faces |   1.524459   .3299328     1.95   0.051     .9974559    2.329904
-------------+----------------------------------------------------------------
       theta |    3.36901   .8302831
------------------------------------------------------------------------------
LR test of theta=0: chibar2(01) = 73.65                Prob >= chibar2 = 0.000

Note: Standard errors of hazard ratios are conditional on theta.

. stcox RealizaçãodeForramento shared (Numero_do_Paciente)
variable shared not found
r(111);

end of do-file

r(111);

. do "C:\Users\thiar\AppData\Local\Temp\STD00000000.tmp"

. stcox RealizaçãodeForramento, shared (Numero_do_Paciente)

         failure _d:  Condicao_Restauracao
   analysis time _t:  Idade_da_restauracao

Fitting comparison Cox model:

Estimating frailty variance:

Iteration 0:   log profile likelihood = -826.48785
Iteration 1:   log profile likelihood = -826.24759
Iteration 2:   log profile likelihood = -826.24709
Iteration 3:   log profile likelihood = -826.24709

Fitting final Cox model:

Iteration 0:   log likelihood = -940.69069
Iteration 1:   log likelihood = -855.63659
Iteration 2:   log likelihood = -828.66333
Iteration 3:   log likelihood = -826.31372
Iteration 4:   log likelihood = -826.24755
```

```
Iteration 5:   log likelihood = -826.24709
Iteration 6:   log likelihood = -826.24709
Refining estimates:
Iteration 0:   log likelihood = -826.24709

Cox regression --
         Breslow method for ties                Number of obs     =      2,405
         Gamma shared frailty                   Number of groups  =        348
Group variable: Numero_do_~e
                                                Obs per group:
No. of subjects =        2,405                                min =          1
No. of failures =          122                                avg =  6.9109195
Time at risk    =        19386                                max =         27

                                                Wald chi2(1)      =       0.00
Log likelihood  =   -826.24709                  Prob > chi2       =     0.9856

------------------------------------------------------------------------------------
-----
                    _t | Haz. Ratio   Std. Err.      z    P>|z|     [95% Conf.
Interval]
-----------------------+------------------------------------------------------------
-----
RealizaçãodeForramento |   .9947354    .290812    -0.02   0.986     .5608643
1.764239
-----------------------+------------------------------------------------------------
-----
                 theta |   3.241233    .806649
------------------------------------------------------------------------------------
-----
LR test of theta=0: chibar2(01) = 71.55              Prob >= chibar2 = 0.000

Note: Standard errors of hazard ratios are conditional on theta.

. stcox i.Setor_da_Cidade0, shared (Numero_do_Paciente)

         failure _d:  Condicao_Restauracao
   analysis time _t:  Idade_da_restauracao

Fitting comparison Cox model:

Estimating frailty variance:

Iteration 0:   log profile likelihood = -809.07432
Iteration 1:   log profile likelihood = -808.96615
Iteration 2:   log profile likelihood = -808.96432
Iteration 3:   log profile likelihood = -808.96432

Fitting final Cox model:
```

```
Iteration 0:   log likelihood = -928.32655
Iteration 1:   log likelihood = -833.43835
Iteration 2:   log likelihood = -810.64875
Iteration 3:   log likelihood = -809.00386
Iteration 4:   log likelihood = -808.96454
Iteration 5:   log likelihood = -808.96432
Iteration 6:   log likelihood = -808.96432
Refining estimates:
Iteration 0:   log likelihood = -808.96432

Cox regression --
         Breslow method for ties                Number of obs     =      2,405
         Gamma shared frailty                   Number of groups  =        348
Group variable: Numero_do_~e
                                                Obs per group:
No. of subjects =        2,405                                min =          1
No. of failures =          122                                avg =  6.9109195
Time at risk    =        19386                                max =         27

                                                Wald chi2(4)      =      31.10
Log likelihood  =   -808.96432                  Prob > chi2       =     0.0000

------------------------------------------------------------------------------------
             _t | Haz. Ratio   Std. Err.      z    P>|z|     [95% Conf. Interval]
----------------+-------------------------------------------------------------------
Setor_da_Cidade0 |
            sul  |   .0397766   .0283242    -4.53   0.000     .0098515    .1606025
          leste  |   .2411774   .1388806    -2.47   0.014     .0780149    .7455829
          oeste  |   .2804832   .0878877    -4.06   0.000     .1517707    .5183531
         centro  |    .138436    .093391    -2.93   0.003     .0368991    .5193766
----------------+-------------------------------------------------------------------
          theta |   2.410315   .6262277
------------------------------------------------------------------------------------
LR test of theta=0: chibar2(01) = 58.35               Prob >= chibar2 = 0.000

Note: Standard errors of hazard ratios are conditional on theta.

. stcox Espcialidade, shared (Numero_do_Paciente)

         failure _d:  Condicao_Restauracao
   analysis time _t:  Idade_da_restauracao

Fitting comparison Cox model:

Estimating frailty variance:

Iteration 0:   log profile likelihood = -826.43369
Iteration 1:   log profile likelihood =  -826.1538
Iteration 2:   log profile likelihood = -826.15281
Iteration 3:   log profile likelihood = -826.15281
```

```
Fitting final Cox model:

Iteration 0:   log likelihood = -941.34069
Iteration 1:   log likelihood = -856.10041
Iteration 2:   log likelihood = -828.61684
Iteration 3:   log likelihood = -826.21971
Iteration 4:   log likelihood = -826.15329
Iteration 5:   log likelihood = -826.15281
Iteration 6:   log likelihood = -826.15281
Refining estimates:
Iteration 0:   log likelihood = -826.15281

Cox regression --
         Breslow method for ties                Number of obs     =      2,405
         Gamma shared frailty                   Number of groups  =        348
Group variable: Numero_do_~e
                                                Obs per group:
No. of subjects =       2,405                                 min =          1
No. of failures =         122                                 avg =  6.9109195
Time at risk    =       19386                                 max =         27

                                                Wald chi2(1)      =       0.19
Log likelihood  =   -826.15281                  Prob > chi2       =     0.6612

------------------------------------------------------------------------------
          _t | Haz. Ratio   Std. Err.      z    P>|z|     [95% Conf. Interval]
-------------+----------------------------------------------------------------
Espcialidade |   .8787263   .2591867    -0.44   0.661     .4929303    1.566469
-------------+----------------------------------------------------------------
       theta |   3.289396   .8191302
------------------------------------------------------------------------------
LR test of theta=0: chibar2(01) = 70.60              Prob >= chibar2 = 0.000

Note: Standard errors of hazard ratios are conditional on theta.

. stcox temponovo, shared (Numero_do_Paciente)

         failure _d:  Condicao_Restauracao
   analysis time _t:  Idade_da_restauracao

Fitting comparison Cox model:

Estimating frailty variance:

Iteration 0:   log profile likelihood = -821.57428
Iteration 1:   log profile likelihood = -821.54644
Iteration 2:   log profile likelihood = -821.54643

Fitting final Cox model:
```

```
Iteration 0:   log likelihood = -935.71606
Iteration 1:   log likelihood =  -849.0246
Iteration 2:   log likelihood = -823.55077
Iteration 3:   log likelihood =  -821.5933
Iteration 4:   log likelihood = -821.54672
Iteration 5:   log likelihood = -821.54643
Iteration 6:   log likelihood = -821.54643
Refining estimates:
Iteration 0:   log likelihood = -821.54643

Cox regression --
         Breslow method for ties                Number of obs     =      2,405
         Gamma shared frailty                   Number of groups  =        348
Group variable: Numero_do_~e
                                                Obs per group:
No. of subjects =        2,405                                min =          1
No. of failures =          122                                avg =  6.9109195
Time at risk    =        19386                                max =         27

                                                Wald chi2(1)      =       8.06
Log likelihood  =   -821.54643                  Prob > chi2       =     0.0045

------------------------------------------------------------------------------
          _t | Haz. Ratio   Std. Err.      z    P>|z|     [95% Conf. Interval]
-------------+----------------------------------------------------------------
   temponovo |   4.106578   2.043301     2.84   0.005     1.548637    10.88956
-------------+----------------------------------------------------------------
       theta |    2.88792   .7359146
------------------------------------------------------------------------------
LR test of theta=0: chibar2(01) = 65.61              Prob >= chibar2 = 0.000

Note: Standard errors of hazard ratios are conditional on theta.

.
end of do-file

. label define Setor_da_Cidade0 0 "com especialidade", modify

. label define Setor_da_Cidade0 0 "norte", modify

. la define Espcialidade 0"espedialista" 1"sem espc"

. la value Espcialidade

. la value Espcialidade Espcialidade

. tab Espcialidade

Espcialidade |      Freq.     Percent        Cum.
```

```
-------------+-----------------------------------
espedialista |      1,710       71.10       71.10
    sem espc |        695       28.90      100.00
-------------+-----------------------------------
       Total |      2,405      100.00

. sum Numero_do_Paciente

    Variable |        Obs        Mean    Std. Dev.       Min        Max
-------------+---------------------------------------------------------
Numero_do_~e |      2,405    174.0948    95.12042          1        351

. save "C:\Users\thiar\Documents\lenovo\Backup
05-02-2020\_disco_D\Arquivos_User\Documents\estati
> sticas para outros\carlos soares\para stata 03-08.dta", replace
file C:\Users\thiar\Documents\lenovo\Backup
05-02-2020\_disco_D\Arquivos_User\Documents\estatisti
> cas para outros\carlos soares\para stata 03-08.dta saved

. log close
      name:  <unnamed>
       log:  C:\Users\thiar\Documents\lenovo\Backup
05-02-2020\_disco_D\Arquivos_User\Documents\e
> statisticas para outros\carlos soares\carlos 06-08 do file.log
  log type:  text
 closed on:   7 Aug 2020, 13:41:08
-----------------------------------------------------------------------------------
--------------
```
